# Herbal Medicine and Acupuncture for Breast Cancer Palliative Care and Adjuvant Therapy

**DOI:** 10.1155/2013/437948

**Published:** 2013-06-12

**Authors:** Guo-Shiou Liao, Maria Karmella Apaya, Lie-Fen Shyur

**Affiliations:** ^1^Tri-Service General Hospital, National Defense Medical Center, Taipei 114, Taiwan; ^2^Agricultural Biotechnology Research Center, Academia Sinica, No. 128, Section 2, Academia Road, Nankang, Taipei 115, Taiwan; ^3^Graduate Institute of Pharmacognosy, Taipei Medical University, Taipei 110, Taiwan

## Abstract

Breast cancer is a life-threatening disease among women worldwide with annual rates of reported incidence and death increasing alarmingly. Chemotherapy is a recommended and effective treatment option for breast cancer; however, the narrow therapeutic indices and varied side effects of currently approved drugs present major hurdles in increasing its effectiveness. An increasing number of literature evidence indicate that complementary and alternative medicine (CAM) used in treatment-related symptom control and alleviation of side effects plays an important role in increasing survival rate and quality of life in breast cancer patients. This review focuses on the use of herbal medicines and acupuncture in palliative care and as adjuvants in the treatment of breast cancer. Herbal medicinal treatments, the correlation of clinical use with demonstrated *in vitro* and *in vivo* mechanisms of action, and the use of certain acupoints in acupuncture are summarized. The aim of this review is to facilitate an understanding of the current practice and usefulness of herbal medicine and acupuncture as adjuvants in breast cancer therapy.

## 1. Introduction

Breast cancer remains to be the leading cause of cancer death among women worldwide with the rate of reported incidence and mortality increasing annually [1, 2]. In the past decade, women with tumors between stages I and II increased from 41% to 65%, 80% of which are invasive tumors originating from ductal carcinoma and its variants [3]. Current early detection methods allow breast cancer to be diagnosed at an early stage when successful treatment is more likely. Multiple agencies and organizations around the world support mammography as the most reliable way to detect breast cancer at an early stage, particularly in women aged 50 years and older [4, 5]. About 70% of breast cancers express estrogen hormone receptor (ER) and/or progesterone receptor (PR), and these markers along with human epidermal growth factor receptor 2 (HER-2) and proliferation marker Ki-67 provide information about tumor grade and possible response to different treatments [6]. Although several treatment options are currently available including surgery, radiation therapy, and chemotherapy, specific treatment strategies depend on characteristics such as tumor grade, hormone receptor status, metastatic potential, and molecular and patient profile [7]. Chemotherapy is still the most commonly used and recommended treatment option for breast cancer, either by using a single compound or combination therapy with multiple drugs [8]. However, chemotherapeutic drugs have narrow therapeutic indices resulting in nonselective toxic effects on normal tissues, thus increasing the risk of infection. Although chemotherapy and radiotherapy are effective against breast cancer, they are accompanied by varied side effects including vasomotor syndrome (occurring in up to 80% of patients), nausea and vomiting (75%), postmastectomy edema (30–60%), arthralgia (over 40%), neutropenia, cachexia, fatigue, pain, hair loss, hot flushes, and psychological stress, which present major hurdles in increasing the effectiveness of cancer therapy [7–10].

Palliative care is an important aspect of cancer therapy that centers on the relief of pain and other symptoms related to cancer and its treatment. It aims to improve the patient's quality of life (QOL) and can be administered along with curative treatment. Pharmacological interventions that reduce or prevent adverse side effects and increase chemosensitivity may have a substantial impact on cancer treatment and palliative care. Though the use of complementary and alternative medicine (CAM) by cancer patients is not part of conventional cancer palliative care regimens in some countries, according to the World Health Organization (WHO), 80% of cancer patients use CAM, in one form or another, for these purposes [11]. According to the WHO definition, the term CAM is used interchangeably with “traditional medicine” and refers to a broad set of health care practices, including traditional Chinese medicine (TCM), acupuncture, herbal preparations, vitamins, homeopathic remedies, music therapy, and other psychological, physical, and spiritual techniques [12–14]. The effectiveness of CAM is primarily based on empirical evidence and case studies however, in the recent years, the increasing amount of supporting data from controlled clinical trials relating CAM use to overall quality of life and safety has dramatically increased [15–17]. These supportive measures are supposed to control symptoms, improve QOL, boost the immune system, decrease cytotoxicity to normal cells, and possibly prolong life [17–20]. It may also be important to note that the integration of CAM into palliative care and cancer treatment regimens is influenced by culture [21]. One multicenter study that reported oncology professionals' attitude towards CAM concluded that in European countries, for example, CAM therapies commonly include mistletoe extracts, vitamin supplementation, and phytoestrogens, and only an approximately 4% of Scandinavian health practitioners, in contrast to the 20% German doctors, believe that CAM use has a positive role in adjuvant treatment of cancer patients [22]. Traditional oriental medicine systems (Chinese, Japanese, Korean, or Ayurvedic), spiritualism, hypnosis, aromatherapy, and acupuncture represent the widespread use of these CAM practices in the region [23]. In particular, China, Japan, Republic of Korea, and Taiwan operate a two-tiered medical system of integrative medicine, and CAM is fully integrated into national health, education, and insurance policies [16, 24, 25]. On the other hand, though not integrated in current oncological practice, 16% to 63% of North American cancer patients are reported to commonly use acupuncture, hypnosis, and spiritualism, as well as vitamin therapies and botanicals. In one population survey, 75% agreed that combining conventional medical treatment and CAM was preferable to using either alone [26, 27]. The apparent widespread use of CAM worldwide and its (erroneous or otherwise) association with minimal or zero risk means that there is a significant need to do further studies to gain an understanding of the pharmacodynamic interactions between chemotherapeutic drugs and herbal components and the effects of either component and dosing regimens in cancer treatment and palliative care [28, 29].

Among cancer patients, CAM is used more frequently by breast cancer patients with an estimated use by 45% of patients across different treatment stages [30, 31]. In one survey done among long-term breast cancer survivors (on average, 8.7 years after-diagnosis), more than 50% believed that CAM use could prevent cancer recurrence (69%), play an active role in recovery (67%), and help to manage stress (64%) [32]. Evidence gathered from recent randomized control trials (RCTs) demonstrates that herbal medicines and chemopreventive phytochemicals in combination with chemotherapeutic agents are effective in sensitizing cancer cells to treatment and minimizing the side effects arising from conventional therapy, thus increasing patient survival rate and QOL [18, 33–40]. In addition to herbal medicine, acupuncture has also become a popular complementary treatment in oncology, particularly as patients seek nonpharmacological alternatives to provide symptom control. A review of recent RCTs of acupuncture in oncology suggests that it has a promising role in controlling a wide variety of cancer and treatment-related symptoms. The evidence currently available suggests that acupuncture is a safe, low cost, and effective therapy, which further permits cancer patients to actively participate in their own care plan [41].

 Several reviews had been done in the past on the use of either herbal medicine or acupuncture [42–47]; however, a comprehensive review of clinical trials utilizing either of these CAM methods in palliative care of breast cancer patients had not been done yet. This review focuses on the use of herbal medicine treatments, either as single herbs or combinations, and acupuncture in palliative care and as adjuvants in combination with chemo- or radiotherapy in the treatment of breast cancer based on recently conducted or completed RCTs. The correlation between clinical use, *in vitro* mechanistic and *in vivo* animal studies of herbal medicine, and the effectiveness of acupuncture with the use of certain acupoints in breast cancer patients is summarized and is aimed at facilitating an understanding of current practices involving the use of herbal medicine and acupuncture as adjuvants in breast cancer therapy.

## 2. Methods

An electronic search for previously published articles was conducted in PubMed, the Cochrane Database, the US National Center for Complementary and Alternative Medicine (NCCAM) (http://www.nccam.nih.gov/), and the US National Institutes of Health (http://www.clinicaltrials.gov/) databases to find relevant studies published up until February 2013 (inclusive). The search included the following specific medical subject heading (MeSH) terms: breast cancer, AND/OR breast neoplasms, AND/OR adjuvant chemo/radiotherapies, AND/OR herbal medicine, AND/OR acupuncture, AND/OR acupuncture points in addition to relevant text keywords comprising the following words in combination: cancer palliative care, traditional Chinese medicine, herbal formulation, phytoagent, and acupoints. The article or study types were limited to clinical trials (Phases I to IV), controlled clinical trials, and randomized controlled trials (RCTs). The titles and abstracts of all retrieved citations were read and analyzed. In total, 90 RCTs, either completed or ongoing, were included regardless of blinding. The most common phytoagents, single herbal treatments, herbal formulations, and acupoints used in the retrieved RCTs were singled out. Moreover, an exhaustive search for references regarding *in vivo* and* in vitro* studies pertaining to mechanistic actions, acupuncture practices, individual acupoints, treatment-related symptoms, and associated effects was conducted.

Furthermore, the herbal medicine and acupuncture practices included in the most recent RCTs are highlighted in this review because these are generally accepted as constituting the most reliable evidence of treatment effects [48, 49]. RCTs include experiments wherein individuals are randomly allocated to receive or not receive experimental preventive, therapeutic, or diagnostic procedure; they are then followed over a given time period to determine the effects. The RCTs included in this review were either completed or ongoing and are assumed to have complied with health and ethics regulations in the countries where they were conducted. In the following sections, we summarize and describe the results and discuss in some detail the related mechanisms of action and therapeutic effects of these CAM practices as applied in the adjuvant treatment and palliative care of breast cancer patients.

## 3. Results and Discussion

### 3.1. Herbal Medicines as Adjuvant Treatment in Breast Cancer Chemotherapy

The most common complaint among patients receiving chemotherapy treatment is fatigue, which is experienced by 80% to 96% of the patient population [50]. Chemotherapy-induced mucositis and myelosuppression, experienced by almost 40% of patients, are the common, dose-limiting, and costly side effects of cancer therapy [51]. Moreover, cytotoxic chemotherapy suppresses the hematopoietic system, impairing the immune system and limiting the doses of drugs that can be tolerated by the patient [52].  Table 1 summarizes the conventional/approved drugs used in breast cancer chemotherapy together with their major mechanisms of action and most commonly observed side effects [50–63]. Several drugs have been used in combination, for example, cisplatin-methotrexate fluorouracil (CMF), fluorouracil-epirubicin-cyclophosphamide (FEC), and FEC-tamoxifen (FEC-T), supposedly to increase efficacy and reduce side effects. Patients, however, still experience fatigue, phlebitis, alopecia, nausea, mucositis, anemia, and myelosuppression alongside long-term side effects including ovarian failure, weight gain, cardiac dysfunction, and, in some cases, leukemia [64, 65]. Herbal medicines and natural supplements are widely used in cancer chemoprevention in the clinic and are also studied *in vitro* and *in vivo* [66, 67]. 

#### 3.1.1. Traditional Chinese Medicines Composed of Multiple Herbs

In traditional medicinal systems, herbal medicines are used often to treat the symptoms associated with cancer and the side effects of cancer treatment [68]. Herbal formulations used in TCM include mixtures of herbal compounds constituted as decoctions, tea, injections, or capsules, which are purported to possess anticancer compounds and are used alone or as adjuvants to existing chemotherapy regimens to improve efficacy and/or reduce drug-induced toxicity [69]. Although TCM is commonly used to counteract the side effects of chemotherapy, scientific evidence for its use in women with breast cancer still is being collected. Among the most common Chinese medicinal herb formulations used in preclinical and clinical practice for breast cancer treatment are Danggui (*Angelica sinensis*-radix) and Ren Shen (*Panax ginseng*-radix), which are reported to have potential beneficial synergistic effects that include decreasing treatment-associated toxicity, psychosocial stress, and fatigue [70]. Jia-wei-xiao-yao-san, commercially known as “Augmented Rambling Powder,” a Chinese medicinal herb formulation containing Danggui, is the most frequently prescribed formula for treating breast cancer and chemotherapy-related symptoms by TCM practitioners in Taiwan. This formulation has a long history of use for alleviation of blood toxicity and sleep disturbance. It is also used to relieve hot flushes and lower serum levels of inflammatory cytokines, IL-6, IL-8, and macrophage protein 1-*β* [71, 72]. 

LSC101, an encapsulated homogenized mixture of dry powdered extracts from a combination of medicinal herbs, including *Astragalus membranaceus*,* Poriae cocos*, *Atractylodes macrocephala*, *Lycium chinense*, *Ligustrum lucidum*, *Paeonia lactiflora*, *Paeonia obovata*, *Citrus reticulata*, *Ophiopogon japonicus*, *Millettia reticulata*, *Oldenlandia diffusa*, *Scutellaria barbata*, *Prunella vulgaris*, and *Glehnia littoralis,* is used widely by breast cancer patients. Its efficacy in attenuating the hematological complications of chemotherapy has been tested in clinical settings [70]. In mouse breast cancer models, the use of LSC101 together with doxorubicin led to significantly higher neutrophil, splenic erythrocyte, and leukocyte counts [71]. In addition, the use of LCS101 together with conventional chemotherapy regimens provided protection against mild to moderate chemotherapy-induced anemia and neutropenia, supporting its use for decreasing hematological toxicity but not cancer prevention [73]. Though it is not yet clear how the compounds in LCS101 reduce hematological toxicity, it is suspected that the interactions and synergistic effects of the active compounds from the combination of herbs may be responsible for the pronounced efficacy [74]. Some of the component herbs, for example, *Ophiopogon japonicus* and *Astragalus membranaceus,* in LSC101 have been independently shown to stimulate the production of erythroid progenitor cells in mice and promote recovery of hematopoietic function in patients with chronic aplastic anemia [75]. A TCM formulation composed of five herbs, commonly known as “Ruyiping” and “Runing II,” is used as treatment for detoxification and preventing relapse, recurrence, and metastasis in breast cancer patients after mastectomy [76]. Clinical evidence suggests that the mechanism of action of this herbal formulation is via inhibition of angiogenesis and downregulation of vascular endothelial growth factor (VEGF) and VEGF receptor as well as microvessel count (MVC) and micro-vessel area (MVA) [75, 76].

Shenqi Fuzheng Injection (SFI), a TCM formulation used in repairing immune function at the cellular and molecular levels, is also effective in alleviating myelosuppression and GI tract reaction induced by chemotherapy and surgical operation [77]. In clinical evaluations, the protein expressions of CD83, CD80, and CD86 in patients' tumor tissue and auxiliary lymph nodes were detected before and after treatment. Results suggest that SFI could help repair immunity impaired by cancer and cancer treatment by activating dendritic cells and upregulating costimulatory molecules [77, 78]. Clinical studies using a natural dietary supplement composed of a combination of medicinal mushrooms *Coriolus versicolor*, *Ganoderma lucidum*, and *Phellinus linteus* and medicinal herbs *Scutellaria barbata*, *Astragalus membranaceus*, and *Curcuma longa* suggested that the formula can alleviate chemotherapy-induced toxicity in liver, spleen, kidney, lung, and heart tissue [79]. *In vitro* studies also elucidated the mechanism of action of this mushroom-herbal formulation in inhibiting proliferation and lowering the invasive behavior of a highly metastatic human cancer cell line, MDA-MB-231, by the inhibition of cyclin A1 expression and by the downregulation of CXCR4 [79–81].

A combination of rose geranium (*Pelargonium graveolens*, Geraniaceae), *Ganoderma tsugae* (Ganodermataceae), *Codonopsis pilosula* (Campanulaceae), and *Angelica sinensis* (Apiaceae) (RG-CMH) has been used in TCM treatments for breast cancer and is associated with immunomodulation based on anti-inflammatory and wound-healing properties attributed to the synergistic activity of the components of the herbs [82]. In one RCT, RG-CHM intervention improved the immune cell count of cancer patients receiving chemotherapy and/or radiotherapy preventing leukopenia and immune impairment associated with a decrease in levels of T cells, helper T cells, cytotoxic T cells, and natural killer cells compared with the group receiving placebo treatment. However, the differences between the two groups were not statistically significant [83]. The results did show, however, that the administration of RG-CMH to patients receiving chemotherapy/radiotherapy delayed the reduction in levels of leucocytes and neutrophils experienced by patients undergoing cancer treatment [83, 84].

Yunzhi-Danshen (*Coriolus versicolor* and *Salvia miltiorrhiza*) capsules have been shown to benefit the circulatory system through vasodilation, immunomodulation, and antidementia activities [85]. Results of a recent RCT showed that the absolute counts of T-helper lymphocytes (CD4+), the ratio of T-helper (CD4+)/T suppressor and cytotoxic lymphocytes (CD8+), and the percentage and the absolute counts of B lymphocytes were significantly elevated in patients after taking Yunzhi-Danshen capsules. These clinical findings imply that regular oral consumption of Yunzhi-Danshen capsules could be beneficial for promoting immunological function in breast cancer patients after chemotherapy [85, 86]. These findings were also supported by *in vitro *results showing that Yunzhi-Danshen treatment inhibited cancer cell proliferation by cell-cycle arrest and downregulation of Akt phosphorylation in MCF7 cells, a human breast cancer cell line, and by inducing apoptosis [87, 88].

#### 3.1.2. Single Herbs and Medicinal Mushrooms

Black cohosh (*Cimicifuga racemosa*) is known in TCM to reduce hot flushes in menopausal women and to have low toxicity. Several clinical studies have backed up this claim [89–92]. Since breast cancer chemotherapeutics such as cytostatics, aromatase inhibitors, or antiestrogens frequently induce or aggravate preexisting menopausal symptoms, extracts of *C. racemosa* are currently being explored as an adjuvant. The mechanisms of action of this phytotherapeutic herb are still not totally understood, but there is growing interest in its usefulness in the treatment of vasomotor symptoms and hot flushes and in preventing the decrease in bone density associated with menopause [93, 94]. The use of black cohosh in clinical trials as an adjuvant to chemotherapy was observed to help patients improve their QOL through relief of vasomotor symptoms [95, 96]. Moreover, *in vitro* studies using MCF7 cells showed the high antitumor activity of *C. racemosa* extracts and their involvement in induction of apoptosis [97].

*Coriolus versicolor* (Yunzhi) also known as *Trametes versicolor* is a popular component in TCM mushroom preparations. Several clinical trials with patients receiving chemotherapy or radiotherapy have found that encapsulated Yunzhi preparations significantly improve appetite, alleviate weakness, anorexia, vomiting, dryness of the throat, and spontaneous or night sweats and pain, increase weight, stabilize white blood cell counts, NK cells, IL-2 levels, and CD4/CD8 ratio, and demonstrate a 9% absolute reduction in 5-year mortality rate [98–100]. Polysaccharide-K (PSK), also known as krestin, is one of the active compounds found in Yunzhi [83]. It is a unique protein-bound polysaccharide, which has been used as a chemoimmunotherapy agent. Several RCTs have demonstrated the efficacy of PSK as an adjuvant in cancer therapy, with positive results seen in the adjuvant treatment of gastric, esophageal, colorectal, breast, and lung cancers. PSK is a biological response modifier (BRM) that improves the ability of cancer patients to fight off tumor progression through different mechanisms, most probably by leukocyte activation, regulation of IFN-*γ* and IL-2 levels, and inhibition of metalloproteinases and other enzymes involved in metastatic activity [101–105]. PSK has further been shown to have antioxidant activity which may allow it to play a role as a normal tissue chemo- and radioprotector when used in combination with adjuvant or definitive chemotherapy and/or radiotherapy in the treatment of cancers and may also enable it to defend the host from oxidative stress [106].

*Ganoderma Lucidum*, also known as Lingzhi, is used in TCM to promote health and increase life expectancy [137]. Clinically, the spore powder is used to treat cancer-related fatigue in breast cancer patients undergoing endocrine therapy. Patients given the treatment reported improved physical well-being, less fatigue, less anxiety and depression, and overall better QOL. Comparative evaluation of TNF-*α*, IL-6, and liver and kidney function before and after interventions showed a statistically significant effect [138]. The wide spectrum of biological effects reported for *G. lucidum* in the prevention of chronic diseases, such as hepatitis, hepatopathy, and hypertension, makes it a viable adjuvant for hepatoprotection in cancer therapy [139]. Among the active compounds present in *G. lucidum* extracts, triterpenoids are one of the main components responsible for the pharmacological activities including immunomodulatory, antioxidative, antimetastatic, and antitumor effects [140]. *In vitro* and *in vivo* assays have revealed that the mixtures of triterpenoids in *G. lucidum* exerted antiproliferative effects by inducing apoptosis and cell-cycle arrest [141].

 Ginseng (*Panax Ginseng*) is one of the most well-known herbal remedies and is used in TCM to proactively promote health, vitality, and longevity. Ginseng is ranked as the fourth top-selling herbal medicine globally [142, 143]. In recent years, ginseng has been included in the pharmacopoeias of Germany, Austria, the United Kingdom, and the United States. In adjuvant breast cancer therapy, ginseng has been used to maintain natural energy, increase physical and psychomotor performance, and improve mood and general health [144, 145]. *In vitro* experiments and *in vivo* animal studies have reported that ginsenosides, a group of bioactive compounds identified in ginseng, have a variety of beneficial effects, including immunomodulatory, antistress, antifatigue, and anticarcinogenic effects [146]. 

Accumulating evidence from epidemiologic, clinical, and laboratory studies has revealed an inverse-relationship between increased intake of green tea (*Camellia sinensis*) and relative risk for breast cancer [147]. Green tea extract polyphenon E (PPE) containing bioactive compound epigallocatechin gallate (EGCG) was supplemented as decaffeinated green tea capsules for 2 months in a double-blind, randomized, and placebo-controlled intervention study. Results suggest the beneficial effects of EGCG on LDL-cholesterol concentrations and glucose-related markers [148]. Since green tea has been associated with weight control and cardiovascular disease prevention, its effect on weight gain after breast cancer treatment was also investigated [149]. A slight reduction in body weight and improved HDL and glucose homeostasis was seen in overweight breast cancer survivors. These clinical findings, together with substantial *in vitro* and *in vivo *evidence, suggest that tea polyphenols can be used as chemopreventive agents and as adjuvant treatments for breast cancer [149–151].

Mistletoe (*Viscum album*) extracts have been used for cancer therapy since the early 1920s, most commonly in central Europe [152, 153]. Most recent clinical studies have focused on the use of mistletoe extracts as adjuvants for chemotherapy specifically for nausea/vomiting and the side effects of systemic therapy [154]. The active compounds in mistletoe treatment are the recently identified mistletoe lectins (ML I, II, and III) that consist of two polypeptide chains: a carbohydrate-binding B chain that can bind to cell surface receptors enabling the protein to enter the cell, and the catalytic A chain, which can subsequently inhibit protein synthesis, due to its ribosome-inactivating properties [155, 156]. Other pharmacologically relevant compounds found in mistletoe are viscotoxins and other low molecular proteins, oligo- and polysaccharides, flavonoids, and triterpene acids, which have been found to act synergistically resulting in the cytotoxic and apoptosis-inducing effects of the whole plant extract [157, 158]. One RCT showed that mistletoe preparations boosted the immune system in low doses, helping to improve the QOL and survival of some cancer patients by as much as 40% alongside cotreatment with chemo- and radiotherapy. These results are attributed to the overregulation of genes responsible for immune defense, stress response, apoptosis, and cell-cell adhesion pathways [159–162]. 

*Rhodiola algida* is widely used in TCM to stimulate the immune system. Oral ulcerative mucositis, a common adverse effect of mainstream cytotoxic drugs, limits the nutritional intake of cancer patients. One clinical study demonstrated the effects of *R. algida* in alleviation of the occurrence of oral ulcers after four cycles of chemotherapy using 5-fluorouracil, epirubicin and cyclophosphamide, and postmasectomy [163]. Lymphocyte proliferation was induced and serum levels of IL-2, IL-4, and granulocyte-macrophage colony-stimulating factor (GMC-SF) were increased by taking *R. algida* extracts. While white blood cell (WBC) levels returned to the normal range a week after every cycle of chemotherapy, WBC count increased faster in patients using *R. algida*. Patients also presented fewer and smaller oral ulcers and no liver or renal complications were observed in any of the patients involved in the study. Thus *R. algida* has the potential to be used concurrently with chemotherapy to alleviate the occurrence of oral ulcers [163–165].

Several flavonoids with cytotoxic activity have been isolated from the aqueous extract of the aerial part of *Scutellaria barbata*. Despite identification of several active chemical compounds, none demonstrated more potent cytotoxic activity than the whole plant extract. Thus, the whole herb extract is being used and studied clinically [166–168]. In one multicenter, open-label, and dose-escalation phase 1B clinical trial, *S. barbata* extract was administered orally, once or twice daily on a continuous basis to women with advanced metastatic breast cancer (MBC) receiving chemotherapy. Dose-limiting side effects were decreased including aspartate transferase (AST) elevation, diarrhea, fatigue, and pain, proving this herb to be effective and safe and thus showing promise in the treatment of side effects related to the treatment of women with MBC [169]. Most notably, the components of the whole herb extract work in synergy to inhibit cell proliferation, induce cell-cycle arrest, stimulate ROS production and hyperactivation of poly(ADP-ribose) polymerase(PARP), and inhibit glycolysis [170].

Curcumin, the principal active component of turmeric (*Curcuma longa*), has potential therapeutic activities against breast cancer through multiple signaling pathways [171]. It has been widely reported to reverse chemoresistance and sensitize cancer cells to chemotherapy and targeted therapy in breast cancer [172, 173]. In cell models, curcumin could suppress expression of progrowth and antiapoptosis molecules, induce inactivation of NF-*κ*B, Src, and Akt/mTOR pathways, and downregulate the key epigenetic modifier EZH2 [174–178]. One clinical study reported that when curcumin was used as an adjuvant with docetaxel, dose-limiting toxicity effects were significantly decreased [175]. 

*Uncaria tomentosa*, commonly known as Utor Cat's Claw, is a medicinal herb used in the treatment of different diseases including cancer, arthritis, gout, and epidemic diseases [179]. Whole plant extracts were reported to have cytostatic and anti-inflammatory activity, and patients who use Cat's Claw along with chemotherapy and radiation report fewer adverse effects [180]. The use of *U. tomentosa* helps in the restoration of cellular DNA, preventing mutations and cell damage caused by chemotherapy drugs [181]. In addition to its antioxidant properties, *U. tomentosa* modulates the activity of the immune system by proliferation of normal T and B lymphocytes and modulation of certain cytokines, including IL-1, IL-6, and TNF-*α* [182–184]. 

### 3.2. Use of Acupuncture in Breast Cancer 

#### 3.2.1. Definition and Concept of Acupuncture

The National Institutes of Health (NIH), USA, has defined acupuncture as a family of procedures involving stimulation of anatomical locations on the skin by a variety of techniques. The most studied mechanism of stimulation of acupuncture points uses penetration of the skin by thin, solid, and metallic needles, which are manipulated manually or by electrical stimulation [185].

The general theory of acupuncture is based on the premise that bodily functions are regulated by an energy called “qi” which flows through the body; disruptions of this flow may cause disease [186]. Traditional acupuncturists understand qi as circulating between the organs along channels called meridians, which are classified as yin or yang meridians [187]. Yin meridians include the lung, spleen, heart, kidney, pericardium, and liver, while yang meridians include the stomach, large intestines, small intestines, bladder, triple energizer, and gall bladder [188, 189]. Qi energy must flow in the correct strength and quality through each of these meridians and organs for health to be maintained. Throughout the history of Chinese medicine and descriptions in *Huangdi Neijing*, the concept of balancing yin and yang had been extensively applied in the application of combination of meridians, corresponding organs, and acupuncture points or acupoints [188, 189]. Acupoints are mainly (but not always) found at specific locations along the meridians which provide one means of altering the flow of qi. There are also a number of acupuncture points with specified locations outside the meridians; these are called “extraordinary” points and are often credited with special therapeutic properties. A third category of acupuncture points called “A-shi” points have no fixed location but represent tender or reflexive points appearing in the course of pain syndromes [188]. Acupuncture points are thought to correspond to conventional (Western) physiological and anatomical features, such as the peripheral nerve junctions, and are known to stimulate the release of neurotransmitters, partially explaining its effect particularly in pain management [189]. 

#### 3.2.2. Scientific Exploration into Acupuncture

Acupuncture is aimed at correcting imbalances in the flow of qi by stimulation of acupoints by a variety of techniques which involves the insertion of fine needles into the skin and underlying tissues at specific points, for therapeutic or preventative purposes. Evidence of the neurophysiological mechanisms underlying acupuncture now exists [190–193]. For example, the release of a number of endogenous substances including *β*-endorphin, met-enkephalin, and dynorphins was observed during treatment [194–196]. Moreover, acupuncture can alter gene expression, upregulating opioid production [197, 198]. Acupuncture works by modulating noradrenergic and serotonergic pathways to give extra segmental pain relief, that is, analgesia throughout the body [199]. Acupuncture releases serotonin [200], oxytocin [201], and endogenous steroids [202], which may further contribute to analgesia. In functional MRI studies, acupuncture induced brain activation in the hypothalamus and nucleus accumbens and deactivated areas of the anterior cingulate cortex, amygdala, and hippocampus. In terms of analgesia, it was suggested that acupuncture modulated the affective-cognitive aspect of pain perception [199]. Furthermore, correlations between signal intensities and analgesic effects have been reported [203]. Further work using PET scanning showed that acupuncture induced extra effects in the ipsilateral insula beyond the sham needle, which also had greater effects on activation patterns than the control group [107]. 

Recent advances in clinical research on acupuncture suggest that acupuncture provides clinical benefit for breast oncology patients in symptom control and supportive care. Symptoms that respond to acupuncture treatment include pain, gastrointestinal side effects, hot flushes, fatigue, anxiety, depression, and insomnia. Patients welcome a supportive therapy that can reduce symptoms without the need for long-term medication. The strength of current scientific evidence has made acupuncture more acceptable to Western-trained doctors and given rise to Western medical acupuncture [108].

### 3.3. Effectiveness of Acupuncture in Breast Cancer Patients and Suggested Acupoints

#### 3.3.1. Cancer-Related Hot Flushes

Twelve trials explored the effect of acupuncture on vasomotor syndrome (summarized in Table 2) [109–116, 204, 205], including eight RCTs and four single-group pre-post comparisons. Daily flush frequency was the main outcome measure. All the studies used self-administrated questionnaires to measure this effect. Some trials also used the Kupperman Index (KI) to score climacteric symptoms. Most studies used six or more acupoints of which SP6 was the most commonly used. A course of acupuncture treatment has been found to reduce hot flushes associated with normal menopause and also from hormonal treatments for cancer. Studies found that acupuncture reduced hot flushes by up to 60% in women treated with tamoxifen for breast cancer [108, 110, 111, 205]. Those in the acupuncture group additionally reported improved libido, increased energy, and improved clarity of thought and sense of well-being. Furthermore, the acupuncture group reported no adverse side effects. An algorithm has been developed for the long-term treatment of hot flushes, with the observed effects of the initial course of treatment maintained for up to 6 years by weekly self-needling at SP6 or by using semipermanent needles [117]. For self-needling, patients require clear demonstration of cleansing, insertion, and safe disposal [118].

#### 3.3.2. Nausea and Vomiting

Ten studies included in Table 2 investigated the antiemetic effect of acupuncture on distress symptoms induced by chemotherapy [119–126]. Participants received intervention over a treatment period of 5 days to 3 weeks. These studies, including three high quality studies [118, 119, 121], reported that acupuncture could significantly improve emesis caused by breast cancer therapy. Acupuncture stimulation at points PC6 and ST36 has repeatedly been shown to be a clinically useful antiemetic treatment for postoperative nausea and vomiting and chemotherapy-induced emesis. In 1998, the US NIH stated that “acupuncture is a proven effective treatment modality for nausea and vomiting” [185]. A three-arm RCT comparing conventional antiemetics alone with antiemetics plus either electroacupuncture or minimal acupuncture demonstrated that the electroacupuncture plus antiemetics arm was the most effective for preventing nausea and vomiting associated with high-dose chemotherapy [121]. Ezzo and colleagues reviewed eleven trials in 2006 and concluded that electroacupuncture has demonstrated benefit for chemotherapy-induced acute vomiting, and self-administered acupressure appears to have a protective effect against acute nausea and can readily be taught to patients [206]. Since then, two multicenter longitudinal RCTs have shown the beneficial effect of acupressure in significantly reducing the severity of both acute and delayed vomiting [118, 119]. These studies also demonstrate that acupuncture and acupressure are simple to administer and merit wider consideration. 

#### 3.3.3. Pain

Up to 70% of cancer patients still suffer significant pain which adversely impacts their QOL [207]. Bone pain is the most common type of cancer-associated pain, and bone metastases are common in advanced breast cancers. Current pain-relieving strategies include the use of opioid-based analgesics, bisphosphonates, and radiotherapy. The pharmacological failure to control pain alone has led to the use of nondrug treatments including acupuncture. The analgesic effects of acupuncture may permit a decrease in the requirement and side effects of pharmaceuticals. It can also help those who are sensitive to normal doses of analgesics and those who have pain despite analgesic dose titration [196]. Although acupuncture is used in palliative care settings for all types of cancer pain, the evidence base is still insufficient and inconclusive and there is very little evidence to show its effectiveness in relieving cancer-induced pain [208, 209]. 

 Three trials used acupuncture to manage postmastectomy pain (Table 2) [127–129, 208, 209]. Acupoint L14 was used in all the three trials. Two studies demonstrated a significant effect favoring the acupuncture group [128, 129], but one high quality RCT [127] found no significant difference between the intervention group and the control group. Although reviews vary in their conclusions, acupuncture was found to be superior to no treatment or waiting list control in most studies.

 Finally, emerging evidence demonstrates the analgesic effectiveness of both acupuncture and electroacupuncture in breast cancer patients experiencing joint pain as a result of adjuvant aromatase inhibitor treatment. Four trials have included investigation of arthralgia, and all explored the effect of acupuncture therapy on aromatase inhibitor-related joint pain and functional ability (Table 2). Positive results were obtained including enhanced postoperative analgesic efficiency, relief of postoperative pain, and significant improvement in joint and muscle stiffness [128, 130–132, 210].

#### 3.3.4. Fatigue

Fatigue is an extremely common symptom in cancer patients [211]. Fatigue is also an adverse side effect of chemotherapy and radiotherapy, which can persist long after the cessation of treatment. In a prospective phase II study on patients with persistent fatigue who had previously completed chemotherapy, acupuncture resulted in a significant reduction in baseline fatigue scores [212]. Further four RCTs showed that acupuncture was associated with a significant improvement in general fatigue scores [213–216].

#### 3.3.5. Anxiety, Depression, and Insomnia

The two anxiety, sickness, and dyspnoea (ASAD) points located at the upper left and right sternal regions are used extensively in the UK to control dyspnea and also anxiety. Patients can massage acupuncture studs for 1-2 min on demand to provide anxiolysis [196]. This method has the added benefit of empowering the patient to control these distressing symptoms in the event of a panic attack. In a systematic review of RCTs of acupuncture in the treatment of depression, Leo and Ligot Jr. stated that although the odd ratios derived from comparing acupuncture with control conditions in the existing literature suggest a role for acupuncture, the evidence is thus far inconclusive [217]. More recent evidence suggests that acupuncture when combined with antidepressant therapy has a faster therapeutic onset rate than pharmacotherapy alone, coupled with a reduction in the side effect profile of the antidepressant medication [218]. An additional RCT examining the treatment of hot flushes revealed that compared to women taking venlafaxine, those receiving acupuncture felt they had more energy, improved clarity of thought, increased libido, and a greater sense of well-being [219]. In one study done by Mehling and colleagues, massage and acupuncture in postoperative cancer patients who were also receiving usual care resulted in a significant improvement in their depressed mood with short-lived significant improvement in tension and anxiety when compared to patients receiving usual care alone [220]. A subsequent meta-analysis revealed that the rate of improvement in insomnia produced by auricular acupuncture was significantly higher than that achieved by taking Diazepam [221]. Although a Cochrane systematic review of acupuncture for insomnia in 2007 concluded that acupuncture or its variants were not more effective than the control groups [222], five clinical studies showed significant improvement in anxiety and depression over time in patients who underwent acupuncture treatment. QOL measures of pain severity and interference, physical and psychological distress, life satisfaction, and mood states also showed improved scores after acupuncture treatment [223–227]. 

#### 3.3.6. Lymphoedema and Leukopenia

Lymphoedema is a distressing problem that affects many women after breast cancer surgery. In the United States, needling and even lifting objects using the affected arm has been prohibited, resulting in a limited number of publications on acupuncture and lymphoedema [133]. However, recent results of two studies demonstrated that traditional acupuncture after breast cancer surgery was associated with improvements in movement amplitude of the shoulder, symptoms of heaviness and tightness in the arm, and the degree of lymphedema [133, 134].

Two trials conducted in China found that dexamethasone injected at the ST36 intra-acupoint was effective in preventing bone marrow suppression-related leukopenia in breast cancer patients undergoing chemotherapy or radiotherapy (Table 2) [135, 136]. The main body of evidence comes from China where a systematic review of RCTs was positive for increasing WBC in patients undergoing chemotherapy [228]; however, the quality of trials was considered poor, and the authors suggest that the positive meta-analysis should be considered as exploratory. 

### 3.4. Safety of Acupuncture

With an increasing number of positive evidence-based acupuncture trials, more cancer patients may seek acupuncture treatment. While closely monitored clinical trials often report low incidences of adverse events of acupuncture, many physicians remain concerned about its safety. Serious adverse events are exceedingly rare—roughly five in one million [229]—and are usually associated with poorly trained, unlicensed acupuncturists [230]. The vast majority of adverse events from acupuncture are minor; those most commonly reported occur at the site of needle insertion: minor bleeding (3%), hematoma (2-3%), and pain from needling (up to 3%). Dizziness is reported in about 1% of treatments [229, 231, 232]. Serious adverse effects including pneumothorax, spinal lesions, and hepatitis *B* transmission have been reported in the literature for acupuncture, but these are rare and are generally associated with poorly trained unlicensed acupuncturists [233]. Acupuncture for oncology should be administered by a suitably qualified practitioner who can maintain a constant dialogue with the oncology team treating the patient. The contraindications and cautions for acupuncture in an oncology setting are outlined in Table 3. 

In general, acupuncture can be considered a safe method of treatment, with a low side effect profile, which in part adds to its popularity among patients [234–236]. Establishing an eligibility guideline for cancer patients before receiving acupuncture would add another layer of safety. Lu and Rosenthal suggested that cancer patients should not be recommended for acupuncture if they have one of the following conditions: (a) absolute neutrophil count (ANC) less than 500/*μ*L, (b) platelet count less than 25,000/*μ*L, (c) altered mental state, (d) clinically significant cardiac arrhythmias, and (e) other unstable medical conditions (case-by-case consideration). Guidelines for safe practice within this field have previously been published [237]. Before the first visit, approval is required from the primary oncologist based upon these guidelines [238].

## 4. Conclusions and Future Prospects

Research on CAM as adjuvants in chemotherapy and/or radiotherapy, particularly that on herbal medicine and acupuncture, has gained momentum over the past few years. This development paves the way toward understanding their efficacy and modes of action in alleviating cancer or cancer treatment-related conditions. Evidence from various *in vitro*, *in vivo* studies and RCTs support the use of herbal medicine or acupuncture in boosting the immune system, in relieving pain, fatigue, cyto- and hepatotoxicity, and in inhibiting gastrointestinal toxicity, angiogenesis, and other side effects from chemo- and radiotherapy. The inclusion of selected herbal medicines from well-designed RCTs in this review provides evidence-based knowledge to strengthen the rationale for the use of herbal medicines in controlling breast cancer in the clinical setting. Further, considering the assessment of benefit : risk ratio of the presented results, acupuncture is seen as a valuable nonpharmaceutical treatment option for symptom management in cancer patients. Although current evidence from basic science and clinical research on herbal medicines or acupuncture is still not sufficient to change oncological practice in general, the quality and design of clinical trials have significantly improved over the last few years which can provide patients with the most effective protocols or treatment types and safety profiles. Despite all the evidence presented, key challenges still exist including quality control of herbal medicinal materials, standardization of practices and current methodology in acupuncture, and pharmacokinetic interaction between drug components or between chemotherapy and herbal medicine. Further research addressing these challenges in the form of rigorously designed clinical trials accompanied by comprehensive and in-depth laboratory studies is needed to improve the quality of the existing evidence base and support the use of CAM.

## Figures and Tables

**Table 1:** Summary of major mechanisms of action and common side effects of chemotherapeutic drugs approved for breast cancers.

Chemotherapeutic agent	Mechanism of action	Side effect	References
Cyclophosphamide	Apoptotic cell death	Pulmonary toxicities, weight gain	[50, 52]
Cisplatin	DNA damage, apoptosis	Nephrotoxicity	[54]
Doxorubicin	DNA damage	Impaired cognitive function, anemia	[55, 56]
Docetaxel	Mitotic inhibition	Pulmonary toxicities, colitis, diarrhea	[57]
Epirubicin	DNA damage	Nausea, cardiotoxicity	[58–60]
Fluorouracil	Thymidylate synthetase inhibition; DNA synthesis inhibition	Cardiotoxicity, anemia, GI tract toxicity	[61]
Gemcitabine	Nucleic acid synthesis inhibition	GI tract toxicity	[62]
Methotrexate	Cell cycle arrest	Anemia, weight gain, jaundice, diarrhea, loss of bone density	[50]
Mitomycin	DNA alkylating agent	Myelotoxicity, fatigue, systemic toxicity	[63]
Mitoxantrone	Topoisomerase inhibition	Alopecia, systemic toxicity	[63]

**Table 2:** Summary of the effectiveness of acupuncture (acupoints) used in breast cancer patients for cancer-related syndromes or side effects caused by treatments.

Symptoms	Acupoints used	References
Hot flush	LIV3, GB20, LU7, KI3, SP6, REN4, P7, LIV8 DU14, GB20, BL13, PC7, H6, K7, ST36, SP6, Ear shen men, Ear sympathetic point, BL23, BL32, HT7, SP6, SP9, LR3, PC6, GV20 KI6, SP6, BL23, CV4, GB35, H5 BL62, LR14, KI3, HT7, TE6, SP6, LI11, ST36, GV20, LI4	[107–116]
Nausea and vomiting	P6, ST36, LI4	[117–126]
Postmastectomy pain	LI4, SP6, auricular points, GB6, SJ6, PC2, PC3, LE14, MP19, DI14, BL17, LU2, RE6, RE17	[127–129]
Arthralgia	TB5, GB41, GB34, LI4, ST41, KD3, LI15, SJ14, SI10, SJ4, LI5, SI5, SI3, LI3, Du3, Du8, UB23, GB30, GB39, SP9, SP10, ST34	[130–132]
Lymphedema	CV2, CV3, CV12, LI15, TE14, LU5, TE5, LI4, ST36, ST6, SP9, SJ5, SJ14, REN2, REN3, REN12	[133, 134]
Leukopenia	ST36	[135, 136]

**Table 3:** Contraindications and cautions for the use of acupuncture in breast cancer patients.

	(1) Extreme needle phobia, very “strong reactors” to acupuncture
	(2) Coagulopathy
	(3) Immunocompromised patients or neutropenia (less 500/mm^3^)—risk of infection
	(4) In young patients (age less than 18 years old) postsplenectomy—risk of infection
	(5) Avoid directly onto a tumor nodule or into an ulcerative area
	(6) In areas of spinal instability—risk of cord compression secondary to acupuncture's muscle relaxing properties
	(7) Into a prosthesis—risk of leakage of saline/silicone
	(8) Over intracranial deficits following neurosurgery
	(9) Pregnancy
	(10) Confused patients
